# Post-operative urothelial recurrence in patients with upper urinary tract urothelial carcinoma managed by radical nephroureterectomy with an ipsilateral bladder cuff: Minimal prognostic impact in comparison with non-urothelial recurrence and other clinical indicators

**DOI:** 10.3892/ol.2013.1485

**Published:** 2013-07-23

**Authors:** KIYOSHI TAKAHARA, TERUO INAMOTO, KAZUMASA KOMURA, TOSHIKAZU WATSUJI, HARUHITO AZUMA

**Affiliations:** 1 Department of Urology, Osaka Medical College, Osaka, Japan; 2 Department of Urology, Hirakata City Hospital, Osaka, Japan

**Keywords:** non-urothelial recurrence, upper urinary tract urothelial carcinoma, prognosis

## Abstract

Upper urinary tract urothelial carcinoma (UTUC) is a rare disease, and novel prognostic factors for patients who have undergone a radical nephroureterectomy (RNU) for UTUC have been studied intensely. To the best of our knowledge, the prognostic value of urothelial recurrence in patients with UTUC has not been previously described in studies. The present study compared the prognostic value of urothelial and non-urothelial recurrence in patients with UTUC of the kidney and ureter managed by surgery. The inclusion criteria consisted of a diagnosis of non-metastatic UTUC (any T stage, N0–1 and M0) and receipt of an RNU with an ipsilateral bladder cuff as the primary treatment. Of the 153 patients that were screened for the study, comprehensive clinical and pathological data was available for 103 patients, who were consequently included in the analysis. Overall survival (OS) and cancer-specific survival (CSS) times were estimated. A multivariate analysis was performed using the Cox regression model. The median follow-up period was 29 months (interquartile range, 14–63 months). The patient population was comprised of 71 males (68.9%) and 32 females (31.1%). A total of 32 patients (31.1%) showed non-urothelial recurrence, while 38 patients (36.9%) exhibited urothelial recurrence and 33 patients (32.0%) exhibited no recurrence. When comparing the risk parameters between the non-urothelial recurrence categories, the factors of pathological grade, microvascular invasion, lymphatic invasion and pT classification showed significant differences. However, there were no significant differences between the urothelial recurrence categories. No significant difference was observed between the OS and CSS times within the urothelial recurrence categories (P=0.3955 and P=0.05891, respectively), but significant differences were identified in the non-urothelial recurrence categories (P<0.0001 and P<0.0001, respectively). Among the other relevant descriptive pre-operative characteristics in the multivariate analysis, only non-urothelial recurrence remained associated with a worse CSS [P=0.002; hazard ratio (HR) 9.512]. The results show that urothelial recurrence has a minimal prognostic value in patients with UTUC managed by RNU with an ipsilateral bladder cuff.

## Introduction

Upper urinary tract urothelial carcinoma (UTUC) accounts for ~5% of all urothelial tumors and 10% of all renal tumors (1). Since the disease recurrence and progression rates are high in patients with UTUC (2), an improved understanding of the prognostic parameters may lead to the identification of patients who may benefit from intensified therapy and monitoring.

The classical risk factors for the development of UTUC include smoking, abuse of analgesics, chronic urinary tract infection, urolithiasis and oncological agents, such as cyclophosphamide (3). A significant prognostic factor of UTUC is the disease stage. The five-year survival rate for low stage tumors is ~90%, which decreases to <30% in cases of regional nodal metastases and to <10% in cases of distant metastases (4).

To date, several contemporary, single-center studies of patients who were treated with radical nephroureterectomy (RNU) for UTUC have been published (5–9), and several risk factors for developing UTUC have been reported, including a delay in the RNU (10) and tumor necrosis (11). Although the studies have largely contributed to our understanding of the disease, they were limited by small and heterogeneous populations. To overcome this limitation and to improve our understanding of the natural history of UTUC, a comprehensive database [the Upper Tract Urothelial Carcinoma Collaboration (UTUCC)] incorporating the clinicopathological characteristics and outcomes of >1,300 patients treated with RNU for UTUC at 13 academic centers worldwide was created in 2008 (12). It was concluded that an RNU provided durable local control and cancer-specific survival (CSS) in patients with a localized UTUC, and that the pathological tumor grade, T stage, lymph node status, tumor architecture and lymphovascular invasion (LVI) were significant prognostic variables that were associated with oncological outcomes, which may potentially be used to select patients for adjuvant systemic therapy.

However, there have been no studies that considered the prognostic value of urothelial recurrence in patients with UTUC of the kidney and ureter that is managed by surgery. Consequently, the present study focused on the prognostic impact of urothelial recurrence in comparison with non-urothelial recurrence.

## Patients and methods

### Patient selection

The present study was a retrospective analysis of 153 consecutive patients with UTUC, who underwent surgery between 1996 and 2009 at Hirakata City Hospital (Osaka, Japan). Of the 153 patients that were screened for the study, 103 patients were included in the analysis. The inclusion criteria consisted of a diagnosis of non-metastatic UTUC (any T stage, N0–1 and M0) and receipt of an RNU with an ipsilateral bladder cuff as the primary treatment. No patient had an invasive bladder tumor (BT) at the time of the RNU. Written informed consent was obtained from the patient. This study was approved by the ethics committee of Hirakata City Hospital (Osaka, Japan).

In the past, open RNU using an open excision of the distal ureter with a bladder cuff has been performed to dissect the kidney, with the entire length of the ureter and an adjacent segment of the bladder cuff. From June 2003 to date, the approach that has been used is one of conventional four-trocar nephrectomy. Once the nephrectomy is completed, the ureter is dissected and the intact specimen is moved into the pelvis. Next, a semi-Pfannenstiel incision is made in the lower abdomen, which assists in retrieving the specimen, eases the dissection of the lower ureter and facilitates the excision of the bladder cuff. The hilar and regional lymph nodes that are adjacent to the ipsilateral great vessel are then resected, if possible.

### Study design

The following clinical and pathological variables were evaluated: Gender, age, tumor side, presence of a BT at diagnosis, serum level of C-reactive protein, hemoglobin, histological type, pathological grade, adjuvant chemotherapy, microvascular invasion, lymphatic invasion, urothelial recurrence, non-urothelial recurrence and pathological stage (2002 TNM system). The tumor grading was assessed according to the 1998 World Health Organization/International Society of Urologic Pathology consensus classification (13). All surgical specimens were processed according to the standard pathological procedures at the Hirakata City Hospital. UTUC was defined as a urothelial carcinoma located in the renal pelvis or calices, as well as tumors located within the ureter. C-reactive protein was pre-operatively obtained from the blood of the UTUC patients and collected in a serum-separating tube. The C-reactive protein level was expressed in units of mg/dl. Microvascular invasion was defined as tumor cells in an endothelium-lined space observed using routine light microscopy in whole-mounted UTUC specimens. The oncological follow-up schedule included a physical examination, cystoscopy and CT imaging between the chest and pelvis twice per year during the first five years and annually thereafter.

### Statistical analysis

The continuous parametric variables were reported as mean ± SD and range. The continuous non-parametric variables were presented as median values and interquartile ranges. The F test was used to assess whether the standard deviations of two populations were equal. χ^2^ tests were conducted to assess the differences in the covariate distributions between the urothelial and non-urothelial recurrence categories. CSS was defined as the primary endpoint of the study. The survival interval was defined as the time elapsed between the surgery and the last clinical evaluation or cancer-specific mortality. Survival curves were estimated using the Kaplan-Meier method. The patients who remained alive or succumbed to other causes were censored. The log-rank test was used to compare the survival curves. A Cox proportional hazards regression model was used to verify the clinicopathological variables that independently predicted CSS. In all statistical analyses, a two-sided value of P<0.05 was considered to indicate a statistically significant difference. All data were analyzed using the PASW Statistics version 17 statistical program (SPSS Japan Inc., Tokyo, Japan).

## Results

A total of 103 patients with comprehensive clinicopathological data, who fulfilled the inclusion criteria, were included in the analysis (Table I). The mean age was 68.6 years (interquartile range, 62–75 years). During the follow-up period, 45 patients (43.7%) succumbed to UTUC, 12 (11.7%) succumbed to other causes and 38 (36.9%) displayed evidence of disease recurrence. The median follow-up period for the surviving patients was 29 months (interquartile range, 14–63 months). The tumor was located on the right side in 55 patients (53.4%) and on the left side in 48 (46.6%). A BT was identified at the time of the UTUC diagnosis in 28 patients (27.2%). The histological type was urothelial in 92 patients (89.3%) and non-urothelial in 11 (10.7%). The pathological stage was divided into three groups: Superficial (pT0/pTis/pTa/pT1), muscle-invasive (pT2) and non-organ confined (pT3/pT4), which were identified in 43 (41.8%), 13 (12.6%) and 47 (45.6%) patients, respectively.

The median ages of the patients at the time of the surgery in the groups with non-urothelial recurrence (n=32) and without non-urothelial recurrence (n=71) were 70.5 and 69 years, respectively. The median ages in the groups with urothelial recurrence (n=38) and without urothelial recurrence (n=65) were 69 and 71 years, respectively. When comparing the risk parameters between the non-urothelial recurrence categories, the factors of pathological grade, microvascular invasion, lymphatic invasion and pT classification demonstrated significant differences. However, there were no significant differences observed between the urothelial recurrence categories.

**Figure 1:**
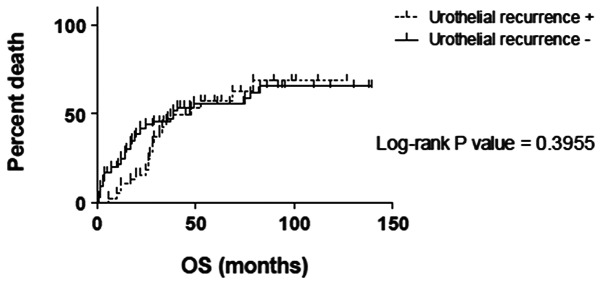
Kaplan-Meier curves of overall survival (OS) time according to the urothelial recurrence categories. Survival curves for the urothelial recurrence-positive (+; broken line) and urothelial recurrence-negative (−; solid line) groups are plotted. The x-axis represents time (months) and the y-axis represents the mortality rate.

**Figure 2:**
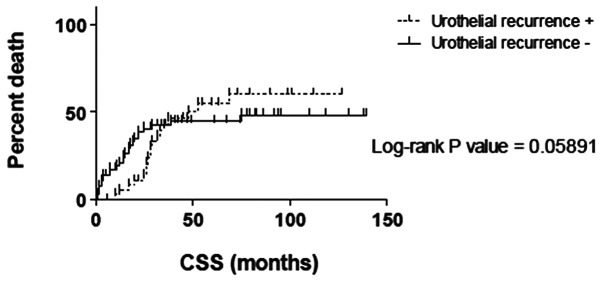
Kaplan-Meier curves of cancer-specific survival (CSS) time according to the urothelial recurrence categories. Survival curves for the urothelial recurrence-positive (+; broken line) and urothelial recurrence-negative (−; solid line) groups are plotted. The x-axis represents time (months) and the y-axis represents the mortality rate. CSS was defined as being alive or succumbing to causes other than cancer. The time period begins at the time of diagnosis and ends at the time of mortality.

**Figure 3:**
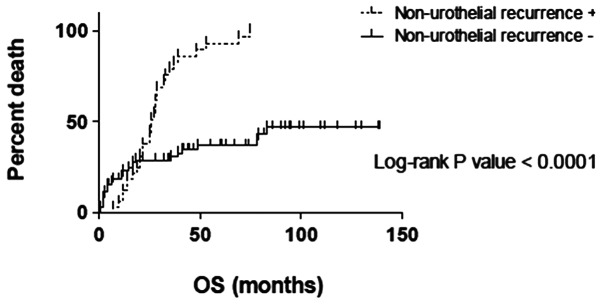
Kaplan-Meier curves of overall survival (OS) time according to the non-urothelial recurrence categories. Survival curves for the non-urothelial recurrence-postive (+; broken line) and non-urothelial recurrence-negative (−; solid line) groups are plotted. The x-axis represents time (months) and the y-axis represents the mortality rate. Results of the log-rank test indicate that OS is significantly higher in the non-urothelial recurrence-negative group (P<0.0001).

**Figure 4:**
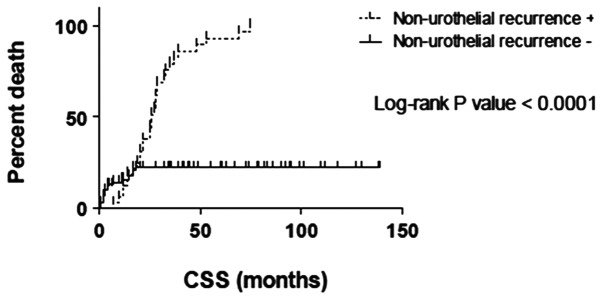
Kaplan-Meier curves of cancer-specific survival (CSS) time according to the non-urothelial recurrence categories. Survival curves for the non-urothelial recurrence-positive (+; broken line) and non-urothelial recurrence-negative (−; solid line) groups are plotted. The x-axis represents time (months) and the y-axis represents the mortality rate. CSS was defined as being alive or succumbing to causes other than cancer. The time period begins at the time of diagnosis and ends at the time of mortality. Results of the log-rank test indicate that CSS is significantly higher in the non-urothelial recurrence-negative group (P<0.0001).

The OS and CSS times between the urothelial recurrence categories showed no significant differences (P=0.3955 and P=0.05891, respectively), while a significant difference was observed within the non-urothelial recurrence categories (P<0.0001 and P<0.0001, respectively; Figs. 1–4).

The univariate analyses using the clinicopathological characteristics, including C-reactive protein, hemoglobin, pathological grade, non-urothelial recurrence, microvascular invasion, lymphatic invasion and pT classification, were associated with the CSS. In the multivariate analysis for the clinicopathological characteristics, only non-urothelial recurrence was associated with a worse CSS (P=0.002, HR 9.512; Table II).

## Discussion

UTUC is a relatively rare malignancy. Although affected patients may benefit from endoscopic or nephron-sparing approaches, an RNU with an ipsilateral bladder cuff excision remains the standard treatment for patients with large, multifocal or high-grade tumors. However, despite definitive surgery, UTUC remains a malignancy with a high potential for local and distant recurrence, particularly in patients with advanced diseases (14). The outcomes of patients with UTUC following an RNU are heterogeneous and, therefore, difficult to predict. Multi-institutional collaborative studies have identified several potential factors that predict the outcome following an RNU for UTUC, supplementing the traditional pathological staging system (15–17).

Certain papers have examined the prognostic value of urothelial recurrence (particularly intravesical recurrence) following the treatment of an RNU for UTUC. Koda *et al* reported that intravesical recurrence following surgery for UTUC was not associated with the mode of surgery (i.e. laparoscopy-assisted or open surgery), and that the only risk factor for intravesical recurrence was a history of bladder cancer (18). Several other studies reported that it may be important to perform careful follow-up appointments that target intravesical recurrence for patients, particularly males and those with low-stage tumors and/or multifocal tumors, following RNU (19, 20). Concomitant carcinoma *in situ* (CIS) and the tumor size were predictors for bladder cancer recurrence (21). In a series of 196 patients, bladder recurrence was lower in those who received mitomycin C or epirubicin compared with those who did not received anything (29.0, 25.9 and 41.3%, respectively) (22). Novara *et al* observed that only a history of bladder cancer prior to an RNU was an independent risk factor for metachronous recurrence, which was identified in 6% of patients (23). Youssef *et al* underlined the prognostic impact of previous bladder cancer (24); patients with a positive bladder cancer (CIS) history had a greater risk of recurrence and mortality from UTUC following RNU (24).

The common locations for the spread of UTUC, depending on the site of the primary tumor, include para-aortic, paracaval, ipsilateral common iliac and pelvic lymph nodes. Hematogenous seeding also occurs in the liver, lungs and bone, which are common sites for metastases. Once a distant metastasis is diagnosed, the prognosis for the patient is extremely poor, in spite of chemotherapy. Certain publications suggest a benefit from the surgical removal of urothelial carcinoma metastases for a subgroup of patients (25). In a large German retrospective study, only 44 patients with distant metastases of the bladder or upper urinary tract underwent a complete resection of all the detectable metastases and were analyzed. The resected metastatic sites included the retroperitoneal lymph nodes (56.8%), distant lymph nodes (11.3%), lung (18.2%), bone (4.5%), adrenal gland (2.3%), brain (2.3%), small intestine (2.3%) and skin (2.3%). Pre- and/or post-metastasectomy systemic chemotherapy was administered in 35 of 44 patients (79.5%). Since no significant prognostic factors were determined due to the limited patient numbers, it was concluded that the metastasectomy in the patients with disseminated urothelial carcinoma metastases remained investigational and there are a limited number of disease types for which a combined-modality approach with systemic chemotherapy would be successful (26).

Lymph node dissection (LND) appears to have an impact on node-positive patients (27). In one previous study, 76 out of 293 patients developed disease relapse. Regional lymph node recurrence was the most common type of relapse (34 patients). In the multivariate analyses that adjusted for the effect of tumor stage and grade, pNx (skipping LND) was an adverse factor for locoregional recurrence and distant relapse (28). However, in the study by Lughezzani *et al*, which analyzed 2,824 patients from the Surveillance, Epidemiology and End Results (SEER) database, LND showed no benefit in patients with an N0 status compared with those with an Nx status (29). Roscigno *et al* suggested that LND should be performed in patients with suspected T2–4 stage diseases, to improve the prediction of the natural history of surgically treated UTUC and to use this information for possible adjuvant chemotherapy (30). Thus, the method of defining the right patient, LND template and the extent of the LND remains unclear.

The present study examined the prognostic value of urothelial and non-urothelial recurrence in patients with UTUC of the kidney and ureter that was managed by surgery. The OS and CSS times between the urothelial recurrence categories showed no significant differences, while significant differences were observed in the OS and CSS between the non-urothelial recurrence categories. The factors of pathological grade, microvascular invasion, lymphatic invasion and pT classification significantly affected the non-urothelial recurrence. However, no factors were observed to significantly affect the urothelial recurrence. In the multivariate analysis for clinicopathological characteristics, only non-urothelial recurrence remained associated with a worse CSS. The present data are limited by the retrospective nature of the study and the relatively small cohort. Prospective studies are required to confirm these findings. However, it may be concluded that non-urothelial recurrence significantly affected the prognosis in patients with UTUC managed by RNU with an ipsilateral bladder cuff compared with those with urothelial recurrence. The findings of the present study underscore the requirement for the careful follow-up and management of urothelial recurrence in patients with UTUC managed by RNU, which aids in lowering the risk of mortality due to cancer.

## Figures and Tables

**Table I:** Clinicopathological characteristics grouped by non-urothelial or urothelial recurrence in 103 patients treated with an RNU and ipsilateral bladder cuff for UTUC.

		Non-urothelial recurrence			Urothelial recurrence		
			
Characteristics	Total	Yes	No	P-value	Yes	No	P-value
Number of patients	103	32	71		38	65	
Gender, n				0.819			0.511
Male	71	23	48		28	43	
Female	32	9	23		10	22	
Age, years				0.788			0.138
Mean	68.6	70.3	67.8		67.3	71.0	
SD	10.1	9.3	10.4		10.2	9.4	
Median	69.0	70.5	69.0		69.0	71.0	
Range	23–91	51–91	23–87		23–87	54–91	
Tumor side, n				0.400			0.221
Right	55	15	40		17	38	
Left	48	17	31		21	27	
BT at diagnosis, n				0.399			0.255
Yes	28	11	17		13	15	
No	75	21	54		25	50	
C-reactive protein (mg/ml), n				0.282			0.432
<0.3	60	16	44		23	37	
≥0.3	43	16	27		15	28	
Hemoglobin (mg/ml), n				0.599			0.614
≤NR	75	14	61		27	48	
>NR	28	18	10		11	17	
Histological type, n				0.166			0.865
Urothelial	92	24	68		32	60	
Non-urothelial	11	8	3		6	5	
Pathological grade, n				0.008			0.278
1	20	3	17		4	16	
2	28	5	23		12	16	
3	55	24	31		22	33	
Adjuvant chemotherapy, n				0.508			0.756
Yes	12	5	7		5	7	
No	91	27	64		33	55	
Microvascular invasion, n				0.001			0.214
Absent	69	13	56		23	46	
Present	34	19	15		15	19	
Lymphatic invasion, n				0.005			0.654
Absent	71	17	54		24	47	
Present	32	15	17		14	18	
pT classification, n				0.001			0.209
pT0/pTis/pTa/pT1	43	6	37		15	28	
pT2	13	2	11		2	11	
pT3/pT4	47	24	23		21	26	

RNU, radical nephroureterectomy; UTUC, upper tract urothelial carcinoma; BT, bladder tumor; NR, normal range; pT0/pTis/pTa/pT1, superficial; pT2, muscle invasive; pT3/pT4, non-organ defined.

**Table II:** Univariate and multivariate Cox regression models for clinicopathological characteristics predicting CSS in 103 patients treated with RNU and ipsilateral bladder cuff for UTUC.

	Univariate			Multivariate		
		
Characteristics	HR	95% CI	P-value	HR	95% CI	P-value
Gender						
Male	Reference		Reference			
Female	0.906	0.467–1.758	0.771	0.945	0.318–2.805	0.919
Age						
Continuous	1.001	0.971–1.033	0.928	0.998	0.922–1.080	0.954
Tumor side						
Right	Reference		Reference			
Left	0.686	0.379–1.241	0.213	0.475	0.172–1.312	0.151
BT at diagnosis						
Yes	Reference		Reference			
No	1.014	0.498–1.952	0.968	2.494	0.089–1.794	0.232
C-reactive protein (mg/ml)						
Continuous	1.184	1.095–1.281	0.000	1.189	0.933–1.516	0.161
Hemoglobin (mg/ml)						
Continuous	1.203	0.732–0.944	0.004	1.133	0.685–1.138	0.337
Histological type, n						
Urothelial	Reference		Reference			
Non-urothelial	2.036	0.924–4.486	0.078	1.951	0.409–9.320	0.402
Pathological grade, n						
1/2	Reference		Reference			
3	4.918	1.984–12.191	0.001	2.288	0.266–19.705	0.451
Adjuvant chemotherapy						
Yes	Reference		Reference			
No	1.183	0.333–2.144	0.723	1.499	0.149–2.989	0.597
Urothelial recurrence						
No	Reference		Reference			
Yes	0.929	0.511–1.690	0.809	0.648	0.215–1.956	0.442
Non-urothelial recurrence						
No	Reference		Reference			
Yes	5.750	3.018–10.954	0.000	9.512	2.293–39.464	0.002
Microvascular invasion, n						
Absent	Reference		Reference			
Present	8.299	3.909–17.618	0.000	3.551	0.585–21.558	0.168
Lymphatic invasion, n						
Absent	Reference		Reference			
Present	3.953	1.966–7.947	0.000	1.205	0.319–4.553	0.784
pT classification, n						
pT0/pTis/pTa/pT1	Reference		Reference			
pT2/pT3/pT4	2.619	1.676–4.092	0.000	0.996	0.275–3.611	0.995

CSS, cancer-specific survival; RNU, radical nephroureterectomy; HR, hazard ratio; UTUC, upper tract urothelial carcinoma; pT0/pTis/pTa/pT1, superficial; pT2/pT3/pT4, muscle invasive/non-organ defined.
